# Supplementary material for: Simulation and team training to improve preterm birth knowledge, evidence-based practices, and communication skills in midwives in Kenya and Uganda: Findings from a pre- and post-intervention analysis
Source: PLOS Glob Public Health. 2023 Jun 8;3(6):e0001695. doi: 10.1371/journal.pgph.0001695 (PMC10249881; doi:10.1371/journal.pgph.0001695)
Supplement: Attachment — Submitted filename: Miller_Reviewer Response_Feb 2023.pdf [file pgph.0001695.s001.pdf]

Miller, Lara

Simulation and team training to improve preterm birth knowledge, evidence-based practices, and communication skills in midwives in Kenya and Uganda: findings from a pre- and post-intervention analysis

**Response to Reviewers**

**Reviewer #1**

---

**In the study population section, it is mentioned that a couple of hospitals were not included in the cluster RCT analysis, can you briefly explain why? And were these hospitals included in this analysis?**
The larger referral hospitals were not included in the cRCT because there not comparable hospitals in the region to serve as control facilities. The line referring the referral facilities not being included in the CRCT was omitted as they were included in this analysis.

**Can you please spell out the acronym AMANAT, or add to the acronym list**
Thank you for pointing out this oversight. AMANAT is a Hindi acronym that translates to Emergency Maternal and Neonatal Care Preparedness. This has been clarified in the manuscript and added to the list of acronyms.

**Was the first knowledge test undertaken before or after the first simulation training in October 2016? I am assuming before, but think it would be worth being explicit about this.**
Before the first simulation training. This has been clarified in the manuscript with the following sentences, "Knowledge tests were administered before the start of and at the conclusion of the intervention. The two time points were: 1) October 2016 (Kenya and Uganda), 2) August 2018 (Uganda) and April 2019 (Kenya)."

**You mention that analyses were done on aggregated data, rather than making comparisons in scores on an individual level. Was this not possible at all, even on a smaller sub-set of people who took part, who were still in post at the end? Appreciate for staff who have left positions etc. they would not be included in an individual analysis, but wondered whether you might be able to analyse a sub-set?**
Thank you for this good point. While we would have like to individual comparisons, we did not collect this individual level data to facilitate this kind of analysis. We were interested in the growth and behavioral change of the facility as a whole and therefore collected only aggregate data for all of the intervention components.

**In the "general communication" questions, can you give a brief example of what kind of things are included?**
An asterisks was added to the table and the following information included, "General communications questions included: thinking out loud, call outs, and other questions related to clear and concise clinical communication."

**I think it is worth briefly commenting upon the impact of the Kenyan strike and the**

**potential impact this had on the training itself and subsequent scores. If the training was repeated once the strike was over (due to many staff leaving) in 2017, this could impact interpretation of the results given that, in this setting, additional training was undertaken – if I have understood the manuscript correctly.**
Thank you for this comment. The national Kenyan nursing strike did have a significant impact on our study. We address this issue in our limitations section and believe that it fully explains the strike and our analysis of how it impacted the study.

**Reviewer #2**

---

**Title**
**I suggest that you put it in the title that this was in the context of a study. Suggest including the study design in the title, ie Simulation training improves pre-term birth knowledge and skills in Kenya and Uganda: results from a cluster randomized controlled trial OR …: findings from a pre- and post- knowledge and skills assessment (since this does not report on the CRCT results but is a sub-analysis)**
The title has been changed to, "Simulation and team training to improve preterm birth knowledge, evidence-based practices, and communication skills in midwives in Kenya and Uganda: findings from a pre- and post-intervention analysis."

**Abstract**
**"Clinical skills related to preterm birth and communication technique used in context were assessed through the use of evidence-based practiced (EBPs) in simulation through StudioCodeTM video analysis." Suggest rewrite to say use of EBPs as recorded on video and analyzed using StudioCode video analysis**
The sentence has been changed to say, "Clinical skills related to preterm birth and communication technique used in context were assessed through the use of evidence-based practiced (EBPs) in video-recorded simulations through StudioCode$^{TM}$ video analysis."

**Use term "controlled trial" rather than "control trial"**
The terms have been updated in the manuscript accordingly.

**What is not clear from the Abstract is that what is being described in this paper is a sub-analysis or an analysis of findings related to training embedded in the CRCT. This is an important distinction because otherwise the reader thinks that this was a CRCT about the training approach alone, rather than a package of interventions of which training was included.**
The following sentence was added to the methods section of the abstract, "This is analysis was nested within the larger CRCT and specifically looked at the impact of the PRONTO portion of the intervention package."

**Key Words**
**Suggest adding Kenya and Uganda to the key words; suggest removing "quality of care" since improved knowledge and skills does not equate improved quality of care**
Kenya and Uganda were added but quality of care was not removed.

## Background

**Overall, the two paragraphs devoted to description of PRONTO training in the Background section seem long as compared to the background and literature cited about the problem of pre-term birth, the known EBPs, and training approaches. Suggest moving the paragraphs supporting PRONTO training to the Discussion to make the background more balanced.**

This section has been trimmed from two paragraphs to one.

**Typo – "dues" should be "due"**

Thank you for catching this, the change has been made.

**Add in statistics about what proportion of neonatal mortality is due to pre-term birth**

We have added the following statement with the corresponding citation, "In both countries, complications related to prematurity account for 40% of neonatal deaths, making it the leading cause of neonatal death; therefore, addressing the clinical care of mothers in preterm labour and babies born preterm is critical."

**The first paragraph lacks sufficient detail. For example, what are the key EBPs which reduce pre-term mortality by 70%? Suggest bring up the list (provision of antenatal corticosteroids for preterm labour, management of preterm babies, and management of neonatal sepsis and**
**pneumonia) to the first paragraph**

This list has been moved to the first paragraph.

**Globally, most maternity and neonatal care is provided by nurses and nurse-midwives with varying levels of training." Training as nurses and nurse midwives is at least standard country by country as these are certified cadres. Perhaps here it should be varying level of skill at EBPs?**

The sentence has been modified to, "Globally, most maternity and neonatal care is provided by nurses and nurse-midwives with varying levels of skills and on-the-job training."

**"Modeling estimates suggest that strengthening the capacity of nurse-midwives to deliver childbirth and post-childbirth services can avert a significant number of stillbirth and neonatal deaths." This statement requires a citation.**

This has been remedied.

**The description of PRONTO should be shortened and authors should consider moving to the Discussion**

The description section has been shortened from two paragraphs to one.

**Overall, the Background section does not adequately describe the placement of the studied training intervention component in the overall PTBi-EA.**

Thank you for this comment. We believe that the following sentences adequately address this point and refer to articles for further reading on the intervention package for interested readers. "To this end, the East Africa Preterm Birth Initiative (PTBi-EA) implemented an intrapartum

package of interventions aimed at improving knowledge and increasing the use of EBPs among facility-based maternity and neonatal care providers, with PRONTO simulation and team training as a key intervention component. The package included data strengthening, a modified version of the WHO Safe Childbirth Checklist, a quality improvement collaborative, and PRONTO."

**Additional citations and more detail is needed on the public health problem of pre-term birth in Kenya and Uganda and the EBTs.**
We have added an additional citation and the following sentence, "In both countries, complications related to prematurity account for 40% of neonatal deaths, making it the leading cause of neonatal death; therefore, addressing the clinical care of mothers in preterm labour and babies born preterm is critical."

**"This pre-post analysis of PRONTO process data from the PTBi-EA CRCT study compares preterm birth knowledge and use of EBPs at the beginning and end of the intervention period." Authors should consider making the design description simpler: ie This study was a pre-and post assessment of knowledge and practice of selected EBPs (list the EBPs assessed) which was embedded in a CRCT. Also, suggest focusing on the dates of the data collected in current study rather than the overall CRCT.**
This change has been made.

**In the Study Population para – suggest removing the (sampling and inclusion/exclusion criteria) as the paragraph does not actually list these. Also, the Levels described here may be country specific – suggest terms such as Regional referral hospital and district hospital may be sufficient.**
These changes have been made.

**Please give more detail on the actual 194 participants trained. How were they selected? Were all health care workers in the 13 facilities trained?**
All were trained. The manuscript sentence has been changed to reflect this, "The study included all of midwives, nurses, clinical officers, and medical officers of the maternity and newborn wards of these facilities."

**Also, how were the 13 facilities selected?**
The following line was added to the manuscript, "Facility sites were chosen for the CRCT based on facilities that had 24 hour labor and delivery service, at least 200 births per year, and had a comparable facility to serve as a control. All thirteen of the study facilities participated in the PRONTO trainings and are included in this analysis."

**Suggest that this section needs two paragraphs: one to define the selection of the facilities in the study and one to define the participants in the study.**
This change has been made.

**"In both countries the pre- and post- intervention groups were not necessarily the same cohort of providers as some nurses may have transferred to other departments, some left or joined the facilities throughout the length of the intervention, or in Kenya the 7-month**

**nursing strike resulted in significant staffing changes throughout their facilities. In addition, in Kenya the mentorship model posed more challenges to sitting and taking a test in the context of providing patient care when needed which resulted in the difference in size of the pre- and post- intervention groups."** This section flags the need to better describe the study design and when the assessments were done in relation to the training intervention. Also, the Sampling section needs to be improved to allow the reader to better understand whose results are being presented. The reader would like to know whether the same 194 providers were assessed pre- and post intervention, and if not, what % of the providers are different? The reader would also like to know in detail when the knowledge assessments took place in relation to the intervention**.**

The following sentence was added to the "Data analysis" section to clarify these points, "Progress was evaluated not by individual performance changes, as individuals working in the facilities fluctuated, but by improvements at the facility and subsequently the country level for both changes in knowledge and EBPs performed in simulations."

**Suggest reducing the term "PRONTO" in describing the intervention. From a reader's perspective, this is a training curriculum for premature infant management. The references to other PRONTO publications are sufficient reminder of the organization behind the training.**

Thank you for this comment and we have considered this point, however the component of the intervention package that referenced to our simulation and mentorship training in partnership with PRONTO International has been referred to as the "PRONTO component" is several other published papers. We have therefore kept the consistent use of terminology in in this manuscript.

(Walker D, Otieno P, Butrick E, Namazzi G, Achola K, Merai R, et al. Effect of a quality improvement package for intrapartum and immediate newborn care on fresh stillbirth and neonatal mortality among preterm and low-birthweight babies in Kenya and Uganda: a cluster-randomised facility-based trial. Lancet Glob Health. 2020 Aug 1;8(8):e1061–70, Namazzi G, Achola KA, Jenny A, Santos N, Butrick E, Otieno P, et al. Implementing an intrapartum package of interventions to improve quality of care to reduce the burden of preterm birth in Kenya and Uganda. Implement Sci Commun. 2021 Dec 28;2(1):10, Otieno P, Waiswa P, Butrick E, Namazzi G, Achola K, Santos N, et al. Strengthening intrapartum and immediate newborn care to reduce morbidity and mortality of preterm infants born in health facilities in Migori County, Kenya and Busoga Region, Uganda: a study protocol for a randomized controlled trial. Trials. 2018;19(313):1–12.)

**Please indicate the timing of the knowledge assessment in relation to the intervention (it is only defined by date of data collection). Authors may consider including this in the design discussion of the Methods.**

This change has been made.

**"All clinical components reflected Ministry of Health guidelines from each country." These are premature care guidelines? Would be good to actually cite these. Would like to hear more about these.**

These additional citations have been added.

**Performance and definition of the EBPs is not sufficiently described in the Methods. Please describe: which EBPs were assessed (perhaps in a table); whether each individual was assessed / recorded separately; and the practical assessment methodology. Was this set up like an OSCE with stations to demonstrate each EBP? Was every participant assessed on every EBP?**

The EBPs evaluated at listed in Table 4. The following sentence was added to the "Data analysis" section, "EBPs were evaluated using a simple binary. The EBP was marked as "performed" if it was seen one or more times in the video of the simulation and "not performed" if it was not." The additional point are addressed with the added sentence in the paragraph above of, "Progress was evaluated not by individual performance changes, as individuals working in the facilities fluctuated, but by improvements at the facility and subsequently the country level for both changes in knowledge and EBPs performed in simulations."

**It would be interesting to note (briefly) why a traditional OSCE assessment was not used for competency assessment and the video assessment was used instead. If not in the Methods, perhaps in the Discussion.**

The following sentence was added to the Background section, "Unlike the traditional Observed Structured Clinical Examination (OSCE) used in this setting, in which examiners use a checklist to observe trainees performing EBPs, PRONTO uses video recording and allows examiners to later watch the videos and carefully identify any problems and provide feedback."

**Suggest combining the sentences about 38 overall questions and 22 questions related to prematurity ie "22 of the overall 38 questions on intrapartum care focused on prematurity and are presented in this analysis"**

This change was made.

**"The presence of preterm birth specific-EBPs were analysed to assess PRONTO's effect on preterm labour, preterm birth, and preterm newborn scenarios." Suggest that this sentence is redundant when placed at the end of the section. If this is sufficiently described in the design section this will not be necessary.**

This sentence was removed.

<u>Results</u>

**Table 3 – more discussion is needed on the attrition rate, which is high – is this described in the Limitations?**

Thank you for this comment, this is addressed in the limitations section.

**Table 3. Why are 324 assessed, when in the Methods it says that 194 were assessed? Please address, either something is not sufficiently described in the Methods or in the Results.**

Thank you for this comment, this was a typo it should read 318 baseline assessments (194 from Kenya and 124 in Uganda) and 233 post-assessment as not all participants remained at the facilities throughout the two years of the program. This change has been madde.

**The column labeled "Simulation(s) where EBP was indicated" is confusing – hard to discern what information is of use for a reader in this column. Suggest dropping this column.**
This column has been removed.

**Table 4 – is this showing the number of people who correctly performed an EBP, or the number of EBPs performed (ie one person could perform multiple EBPs)? Why is the number of people assessed on knowledge and assessed on EBPs so different?**
The labels were changed to more clearly communicate how the EBPs were measured.

**Similarly for the %, it is not clear – is this the % of simulations in which an individual correctly performed the EBP? Or % of simulations where the EBP was performed at all?**
The labels were changed to more clearly communicate how the EBPs were measured.

**Most of this confusion can be resolved by better describing the EBP measurement and procedures in the Methods, and improving the labels on the tables.**
These changes were made.

<u>Discussion</u>

**The number of times PRONTO training is discussed and the circular referencing to other publications about PRONTO training make the article appear to be somewhat promotional about the approach. Since the training PRONTO conducts is based on proven principles and approaches in adult learning and medical / health services education, speaking to the components of the PRONTO approach which are universal – like simulation, team-building, and mentorship – would be more balanced. This would also broaden out the literature included, which would strengthen the paper. For example, if the training were discussed in the context of mentorship, there would be relevant articles in the literature about mentorship approaches in Uganda which could be in the Discussion, similarly simulation.**
Thank you for this comment. We have removed too paragraphs referring to other PRONTO programs and added additional references to other simulation and mentorship training programs in Kenya and Uganda, placing the PRONTO program of PTBi in a larger context of similar programs.

**The Discussion should be clarify that the training described was part of a multi-layer intervention rather than a stand-alone training package.**
We have added additional language making this point clear stating, "As the PTBi-EA intervention package was designed to work synergistically, we cannot specifically separate the impact of PRONTO from the other components of the intervention package (namely, use of a preterm-focused Safe Childbirth Checklist, QI collaboratives, and data strengthening), which also reinforced knowledge and EBPs."

**The discussion could be dramatically improved by comparing the skills building approaches to other skills building approach results, rather than making circular references to other PRONTO trainings**

Thank you for this comment. We have included additional references to other mentorship and simulation-based programs, placing the PTBi PRONTO training in a larger contacts of mentorship and simulation.
