# Supplementary material for: Simulation and team training to improve preterm birth knowledge, evidence-based practices, and communication skills in midwives in Kenya and Uganda: Findings from a pre- and post-intervention analysis
Source: PLOS Glob Public Health. 2023 Jun 8;3(6):e0001695. doi: 10.1371/journal.pgph.0001695 (PMC10249881; doi:10.1371/journal.pgph.0001695)
Supplement: Attachment — Submitted filename: 4MAY23_Miller_Response to reviewers.pdf [file pgph.0001695.s002.pdf]

Response to Reviewers

Abstract

For clearer reading, the authors might consider dropping the "pre-term birth knowledge" and using "knowledge"
This change has been made.

"Clinical skills related to preterm birth and communication technique used in context were assessed through the use of evidence-based practiced (EBPs) in video-recorded simulations through StudioCodeTM video analysis." Is the evidence based practice the use of video recording? Or are the skills documented in the videos compared to EBPs? Not clear from this sentence. Additionally, suggest dropping the 'related to preterm birth' – use of knowledge and clinical skills is sufficient.
These changes were made to read, " Clinical skills and communication technique used in context were assessed through the use of clinical evidence-based practiced (EBPs) as documented in video-recorded simulations through StudioCode^{TM} video analysis."

"Preterm birth-related knowledge assessment test scores improved" this could be more clearly stated by saying "Knowledge scores improved"
This change has been made.

The authors may consider removing the decimal point and simply presenting the whole number % for the knowledge increase
This change has been made.

The authors state that "PRONTO training significantly increased preterm birth-specific knowledge and EBPs performed in simulation." To use the term "significantly increased" I would expect p values or other calculation of statistical significance. Did the authors mean "substantially" or "significantly"?
"Significantly" has been changed to "substantially"

Background

Please check over text for spacing, there are some inconsistencies with too many or too few spaces.
Thank you for bringing this to our attention, the necessary formatting changes have been made.

It is difficult to know what a "proportional" decrease in neonatal mortality would be related to institutional delivery. Did the authors mean resulting or expected decrease? Also, some statistics on institutional births and neonatal mortality rates from the two countries would strengthen this statement.
This sentence has been cut.

"Globally, most maternity and neonatal care is provided by nurses and nurse-midwives with varying levels of skills and on-the-job training." – this statement contains two different assertions. Both could use citations.
This sentence has been cut.

"While PRONTO's efficacy is well-documented, it has yet to be evaluated as a training tool for increasing knowledge and use of EBPs specific to preterm labour and birth. To this end, the East Africa Preterm Birth Initiative (PTBi-EA) implemented an intrapartum package of interventions aimed at improving knowledge and increasing the use of EBPs among facility-based maternity and neonatal care

providers, with PRONTO simulation and team training as a key intervention component. " This statement (erroneously?) suggests that the PTBi-EA was implemented to evaluate PRONTO's efficacy.
This sentence has been cut.

"PTBi-EA demonstrated that the full intervention package resulted in a 34% reduced odds of combined intrapartum and neonatal mortality among preterm infants compared to sites receiving a simplified package of data strengthening and the modified Safe Childbirth Checklist alone." Please put in the actual odds ratio or relative risk from the overview findings. The phrasing of this sentence is not specific enough to understand the effect of the intervention
The odds ratio and confidence intervals have been added.

"To better understand how PRONTO training as an intervention component contributed to the positive effect of the PTBi-EA intrapartum package, we analysed preterm-specific provider knowledge changes and EBPs as performed in simulation pre- and post-invention." This is actually incorrect. The analysis provided does not show how PRONTO training contributed to mortality reduction in the study, ie what part of the mortality reduction was attributable to knowledge and skills gains. This needs to be rewritten.
This sentence has been changed to, "To better understand how the STT impacted preterm birth knowledge and skill of providers in the intervention facilities, we analysed knowledge test changes and EBPs as performed in simulation pre- and post-invention."

The Background section still contains insufficient introduction to the EBPs and that should be improved. It is not in the Methods either. Please add 1 -2 sentences (instead of the half a sentence currently used) Which key EBPs reduce pre-term mortality by 70%? Later in the paragraph, provision of antenatal corticosteroids for preterm labour, management of preterm babies, and management of neonatal sepsis and pneumonia. Are these the EBPs which reduce preterm mortality by 70% and which are assessed in the study? It is not sufficiently clear.
Thank you for this comment. The 70% reference has been removed and the specific EBPs that showed improvements in neonatal mortality in the study referenced included. Additionally, we added a table to the methods section highlighting the appropriate EBPs included in the simulations evaluated in this study.

*The term PRONTO appears 11 times in the Background section. There is no need to repeat the "brand" so many times to explain the training approach. Because the majority of the authors are with PRONTO International, this feels like a promotion of a particular organization or brand. This detracts from the strength of the article. As readers, we are interested in how a training approach which includes simulation, mentorship and use of video to assess competencies, not PRONTO training in particular. I highly suggest that this is addressed. As a reviewer, I cannot make this a required suggestion as there is nothing scientifically incorrect with this approach. However, my strong recommendation is that this is toned down so that the authors do not turn off readers as it is rather off-putting***.**
The authors acknowledge this note and have changed several of the PRONTO references to simulation and team training (STT).

This study was a pre- and post-assessment of knowledge and EBPs performed in simulation embedded in the larger study CRCT. The CRCT data collection began in October 2016 and concluded in August 2018 in Uganda and April 2019 in Kenya (Kenya's timeline was delayed due to the 2017 national nursing strike)." This intro sentence reads much better now – could improve by simply stating that this was embedded in the PTBi cluster randomized controlled trial from 2016 to 2019. The authors provide the citation to the overview paper where readers can get details.
This sentence has been changed

This section has improved since the authors separated out the facilities from the health care providers. However, the section on health care providers: "The study included all midwives, nurses, clinical officers,

and medical officers of the maternity and newborn wards of these facilities" still needs to be substantiated. What we would like to understand in the Methods is: how were health care providers selected (which criteria? All SBAs working in maternity wards?) how were they recruited into the study? And finally, how were they trained? The authors will recall that the Methods should give sufficient detail for other researchers to be able to repeat the study.
The "selection/recruitment" and "training" sections have been expanded to further address these issues. A timeline figure (Figure 1) was also added to further clarify the training activities and when the evaluations were conducted.

Were all providers assessed in knowledge also assessed in EBP using the videography? Or just a subset? Did these assessments take place at the same time and place? Were any of the EBP assessments done in person or all remotely / using videography?
Only a subset were evaluated for skills and only in recorded simulations. This language clarified in the text.

Similarly, the section should be able to describe not only the timing of the assessments in relation to the training, but also who is included in the assessment. The current sentence "In both countries the pre- and post- intervention groups were not necessarily the same cohort of providers as some nurses may have transferred to other departments, some left or joined the facilities throughout the length of the intervention, or in Kenya the 7-month nursing strike resulted in significant staffing changes throughout their facilities." brings up a major challenge to the results presented. Is there any way to quantify the turnover so we can understand how many of the participants actually were exposed to the training? This was brought up in the last round of comments but was not addressed.
Unfortunately, there is no way to quantify the turnover or directly link individual test scores. The study was designed to evaluate at the facility and country level, not the individual level. This is acknowledged as a limitation and discussed in the limitation section.

The term "at country level" should be avoided since these results do not represent the country.
This phrase has been removed.

Please indicate the timing of the knowledge assessment in relation to the intervention (it is only defined by date of data collection).
This is included in the timeline Figure 1.

Performance and definition of the EBPs is not sufficiently described in the Methods. Please describe: which EBPs were assessed (perhaps in a table); whether each individual was assessed / recorded separately; and the practical assessment methodology.
This section has been expanded an additional table (Table 1) added explaining the simulations evaluated and the appropriate evidence-based practiced affiliated with each simulation.

Results

Suggest adding the n of health facilities into the "Kenya" and "Uganda" headings of Table 2
This has been added

FROM FIRST ROUND OF REVIEW, not addressed: Table 3. Why are 324 assessed, when in the Methods it says that 194 were trained? If the study is not measuring the knowledge and skills of those who were trained, how does a reader interpret a gain in knowledge? Was the gain due to other parts of the intervention, not the training? The difference between those trained and more being assessed is not sufficiently described in the Methods or in the Results.

Thank you for bringing this to our attention, 194 participants were trained in Kenya and 124 in Uganda for a total of 318 listed in Table 3. A totals column has been added to Table 2 to clarify this point.

Table 4 is improved! However the table still needs to be clarified further: still needs to be clear the n of EBP assessments and how this is linked with the knowledge assessment. Were they the same people being assessed at the same time? Selected people from the knowledge assessment were assessed on the EBPs? Totally different people? is this showing the number of people who correctly performed an EBP, or the number of EBPs performed (ie one person could perform multiple EBPs)? Why is the number of people assessed on knowledge and assessed on EBPs so different?
Additional language has been added to the methods section on the number of clinicians evaluated per simulation and the total clinicians evaluated added as a note to Table 5 (formerly Table 4).

The column "EBP Indicated for Sim" is not clear. Does this mean that there were 47 video simulations where an EBP was to be performed, and it was performed in 68.1% of them?
This column heading was changed to "No. of sims where EBP was indicated."

Most of this confusion can be resolved by better describing the EBP measurement and procedures in the Methods, and improving the labels on the tables.
Thank you for this comment. We hope at the addition of Table 1 in the methods section and the improved labeling of the tables in the results sections clarifies these points.

Discussion

The discussion could be dramatically improved by comparing the skills building approaches to other skills building approach results, rather than making circular references to other PRONTO trainings. There is much more in the literature about mentorship, simulation, in-service training models in Kenya and Uganda than is mentioned in this Discussion.
Thank you for this comment. Additional discussion around other simulation training programs from Kenya and Uganda has been added to the discussion and a paragraph discussing PRONTO has been cut.

Limitations

"Second, pre- and post- knowledge test scores have disparate sample sizes and are presented as aggregated data per timepoint, rather than as changes on an individual level. While this limits analysis (e.g., evaluating if different cadres of providers differentially benefited from the training), it emphasizes the PRONTO approach of focusing on the clinical team, rather than the individual provider." Was this a deliberate evaluation approach due to the model of training a whole clinical team, or was this a limitation? It seems very much like a limitation to me.
The acknowledgement that this was a limitation of the study in terms of assessing individual provider improvements was clarified.

" Additionally, as mentioned above, the in-situ mentorship model meant there were fewer trainees present for the post-intervention knowledge test than the baseline test ...
Despite this, the fact that significant improvements were still seen in Kenya over the 30-month intervention period speaks to the "rising tide" of PRONTO training" The authors may wish to consider that the fact that skills and knowledge increased when many of those trained were not there in the post-test assessment actually means that other components of the intervention were responsible for the increase in knowledge and skills scores.
Thank you for this comment. The final sentence of this section has been changed to, "Despite this, the fact that significant improvements were still seen in Kenya over the 30-month intervention period speaks to the

strength of the PTBi-EA intervention package as a whole and the reinforcement of best practices through quality improvement work and the WHO Safe Childbirth Checklist. “
